# Can mobile-health applications contribute to long-term increase in physical activity after medical rehabilitation?–A pilot-study

**DOI:** 10.1371/journal.pdig.0000359

**Published:** 2023-10-16

**Authors:** Fabian Borst, Monika Reuss-Borst, Johannes Boschmann, Peter Schwarz

**Affiliations:** 1 HESCURO Clinics, Bad Bocklet, Germany; 2 Department for Nephrology and Rheumatology, Faculty of Medicine Georg-August-Universität Göttingen, Göttingen, Germany; 3 Department for Prevention and Care of Diabetes, Faculty of Medicine Carl Gustav Carus, Technische Universität Dresden, Dresden, Germany; Universität Wien: Universitat Wien, AUSTRIA

## Abstract

Due to the positive effects of rehabilitation declining over time, the aim of this study was to investigate the long-term physical activity level (PAL) following inpatient rehabilitation in relation to the use of a smartphone-based after-care program. 202 patients (mean Body Mass Index (BMI): 30,8 kg/m2; 61% female) with chronic diseases (e.g., diabetes mellitus, obesity, chronic low back pain, depression) were recruited between 08/2020 and 08/2021 in this single-arm observational study. All patients underwent a 3-week inpatient rehabilitation program. PAL (in total activity minutes/week) was measured with a validated (online) questionnaire (Freiburger Questionnaire on PA) after 3, 6, 9, and 12 months. App usage (online time, completion of a course) was recorded automatically and used to evaluate the app user behavior (adherence). A variety of socio-economic factors (age, sex, education level, income etc.) were collected to identify possible barriers of app use. Except for sex, no significant difference was observed for socio-economic factors regarding app usage behavior. Median PAL significantly increased after rehabilitation in the total cohort from 360 min/week (before rehabilitation) to 460 min/week 6 months after rehabilitation, then declined to 420 min/week 9 months after rehabilitation before falling below baseline level after 12 months. There was no significant difference in PAL between app users (45%, 91/202) and non-users (55%, 111/202), although app users tended to retain higher activity levels after 3 and 6 months, respectively. Overall, our study emphasizes the effectiveness of a 3-week rehabilitation program on PAL and the acceptance and usability of a smartphone-based after-care program in this patient group. The adherence to this 3-months after-care app program was acceptable (30%), with modest evidence supporting the effectiveness of app use to sustain PAL in the short term.

## Author summary

Increasing PAL is an important goal of medical rehabilitation in a variety of chronic diseases. This goal is often achieved by inpatient rehabilitation programs but is difficult to implement in everyday life and therefore mostly utilized for short periods of time. In this prospective study, we included 202 patients and followed them over a 12-month period. We recommended patients to use a 3-month smartphone app program at home following a 3-week inpatient rehabilitation, to support them in their transition to everyday life conditions. Use of this smartphone app was recorded automatically and used to evaluate the app usage behavior. Besides sex, we did not find significant differences in socio-economic factors between app users and non-users. In addition, there was no significant difference in PAL (measured by a questionnaire). However, app users tended to be more active after 3 and 6 months respectively, which could indicate an additional short-term benefit of the app program after rehabilitation.

## 1. Introduction

Physical inactivity is one of the most important modifiable risk factors for a variety of chronic diseases, such as rheumatic disorders (osteoarthritis, chronic low back pain), the metabolic syndrome (obesity, diabetes mellitus, gout, arterial hypertension), cardiovascular diseases, some frequent cancers (postmenopausal breast cancer, colon cancer), and also psychiatric diseases, in particular depression [1]. Accordingly, the World Health Organization (WHO) considers a sedentary lifestyle to be one of the four most important risk factors for mortality [2]. Furthermore, physical inactivity is responsible for an enormous economic burden due to health care costs, in addition to indirect costs such as productivity losses and early retirement [3,4]. It is estimated that a global increase in physical activity levels (PAL) would prevent more than 5 million premature deaths per year. Following the WHO recommendations for physical activity (PA) can reduce the risk of various cancers, cardiovascular disease, stroke and diabetes by 20–30% [2]. In addition, there is overwhelming evidence for the positive effects of PA on the course of musculoskeletal diseases, diabetes, obesity, cancer and depression [5–10]. Thus, PA should not solely be considered a preventive measure, as it also acts as a real polypill and may even reduce mortality in cardiovascular and tumor disease by 20–40% [10–12].

Although the positive effects of PA for primary, secondary and tertiary prevention have been known for many years, and the mechanisms by which PA exerts its positive effects on the metabolism, musculoskeletal system and brain have been intensively explored since then, the prevalence of physical inactivity in high-income countries has increased from 32–37% between 2001 and 2016, resulting in it to remaining twice as high as in low-income countries [13–15].

Medical rehabilitation presents an opportunity to initiate a physically active lifestyle by promoting a sustained behavioral change. In Germany, patients with chronic diseases may undergo a three-week inpatient rehabilitation program, which is financed by the German pension fund. A main goal of these programs is to prevent early retirement by initiating a sustainable lifestyle modification. These programs aim to provide support to patients with self-managing the medium- and long-term challenges associated with their chronic conditions. The concept is comprehensive and multi-professional. One main treatment goal is increasing PAL through education, psychological and motivational support, and introduction to physical exercises (endurance and strength training). However, maintaining successful physical activity changes achieved in rehabilitation remains a challenge for most patients.

For these patients, mobile-based applications (apps) may offer an additional, attractive tool to improve long-term outcome after rehabilitation. In recent years, there has been a growing interest in mobile health applications that support health care services through smartphones in everyday life. The number of health-related apps that are published on the two leading platforms (iOS and Android) surpassed 100,000 in 2014, a number which has been exponentially growing since, reaching over 350,000 available digital health applications on app stores in 2021 [16,17]. More than 50% of smartphone users have installed healthcare apps on their smartphones [18–20]. However, most freely available apps are developed outside the healthcare sector, and there typically are no studies on the effectiveness of these tools in changing people’s health-related behaviors. Unsurprisingly, many barriers have been identified that may lead to non-use or high drop-out rates ranging from 23% to 83% [21]. In their recent meta-analysis, for example, Meyerowitz-Katz et al. have shown that up to 80% of all users of mobile Health (m-health) interventions engage only at a minimum level, do not log into the health app more than once, and do not use the app for a longer period [22]. Attrition, defined as lack of patient use of the intervention, is therefore an important issue for mobile interventions. It may stem from technical or individual factors (barriers), such as limited internet skills or low attractiveness of the app (e.g., lack of game-based elements, lack of user-friendly layout, lack of offline functionality, or data privacy concerns) [23]. As a consequence, one observational trial of app usage in a large real-world cohort found that only 2% of participants had sustained continuous use of the kind necessary to improve clinical outcome parameters [24].

In addition, the use of digital interventions may depend on patient-related socio-economic factors such as age, education, and ethnicity. A number of studies suggest that digital health tools should be adopted along with social determinants of health, such as income and education, which may result in a digital health divide, therefore increasing differences in the ability to achieve an improved health outcome due to the use of digital technologies [25,26]. Consequently, this may enhance existing social health inequities, which would not be conducive to current public health efforts [27]. Thus, it seems mandatory to search for barriers of use in m-health technologies in order to develop target-group-specific m-health technologies, with the aim of to improving e-health literacy in populations at risk [26,28].

In summary, there is an urgent need for evidence-based and high-quality m-health applications for rehabilitation after-care programs. This study focuses on the long-term effectiveness on the PAL when participating in a smartphone-based after-care program following rehabilitation [29]. Furthermore, different socio-economic factors, which potentially influence the adherence to this smartphone-based after-care program, are investigated in order to develop target-group-specific post-rehabilitation programs in the future.

## 2. Materials and methods

### 2.1. Study design

This study was designed as a single-arm observational study under real life conditions following the completion of an inpatient rehabilitation program. Eligible patients suffering from chronic diseases (diabetes mellitus, obesity, chronic low back pain, depression) were recruited at the Hescuro-Rehabilitation Center Bad Bocklet (Germany) from August 2020 until August 2021. After an inpatient treatment for 3–5 weeks, patients fulfilling the eligibility criteria were invited to an informational session introducing the voluntary study along with the smartphone application “Videa bewegt”. The app is a certified, digital program, which aims to increase PAL in everyday life using videos combining educational content and training instructions [29,30]. Furthermore, the app contains several additional components to increase motivation and behavioral change, such as goal setting, documentation of progress, messages, and a chat function with the study team. All patients were instructed by the same study personnel.

Inclusion criteria for patients were an age between 18 and 65 years, ownership of a smartphone, adequate German language skills for filling in the questionnaires, and mobility (ability to reach 10,000 steps/day). Exclusion criteria were constant immobility, critically ill patients, and severe psychiatric diseases. The included patients received an identification number (ID) to pseudonymize their personal data. Data protection was described in a data protection concept and monitored by the local data monitoring committee. The study was approved by the ethical committee of the Georg-August University of Göttingen and registered in the German Clinical Trials Register (DRKS00017805).

### 2.2. Study implementation: Rehabilitation and after-care program

In accordance with the legal bases, patients with somatic diseases underwent an intensive inpatient rehabilitation program for 3 weeks and psychosomatic patients for 5 weeks. Based on their primary diagnoses, there were moderate adaptations in content and number of treatments, with all treatment programs being multimodal, combining psychological interventions (psychological counseling, psychoeducation), relaxation techniques (autogenic training, progressive muscle relaxation), health education lessons (e.g., health knowledge, nutritional advice), and exercise therapies including endurance training (ergometry, Nordic Walking, aqua training 2-3x/week) as well strength training (2-3x/week). Treatment was applied according to the German Rehabilitation Treatment Guidelines [31]. A major treatment goal for all rehabilitation patients was to increase their everyday PAL after rehabilitation. This goal was set in accordance with the patients at the beginning of their rehabilitation program. Of the 264 patients invited to participate in the study after their rehabilitation, 202 patients took part in it. Patients were introduced to the app in an information session. Installation assistance was offered as needed. The app granted access to over 40 videos in 8 consecutive courses, providing background knowledge, motivational techniques, and exercises to participate in. The courses were supplemented by quizzes to reinforce the learning outcomes for the patients. In addition, patients were constantly encouraged to take 10,000 steps a day.

### 2.3. Study goals: Physical activity level and socio-economic factors

The primary outcome parameter was PAL, measured as total activity in minutes per week with the “Freiburger Fragebogen zur körperlichen Aktivität” (Freiburg Questionnaire on PAL, FFkA, German version). This is a standardised questionnaire assessing PAL by addressing 12 questions centered around PAL in daily life and leisure time, as well as enquiring about the exercise activity in hours per week [32]. Activity is divided into the following categories: basic activity (e.g., shopping), leisure activity (e.g., taking a walk), and sports activity. Patients were asked to fill out a physical copy of the questionnaire during their rehabilitation (after signing consent) and an online version at 3, 6, 9 and 12 months, respectively. In addition, a variety of socio-economic factors (sex, education level, income) as well as general health-related factors (Body Mass Index (BMI), medical cause for rehabilitation, total physical activity at study start) were documented.

The app program was designed to be completed within 3 months after rehabilitation. Online time, progress, and quizzes were recorded automatically and were used to evaluate the app user behavior (adherence). Additionally, at the end of the study, patients were asked for feedback concerning their adherence, app contents, and possible barriers via an online questionnaire.

### 2.4. Statistical analysis

Socio-economic parameters and their influence on app usage behavior, as well as the changes in long-term activity for different app user types, were tested for significance. As usage behavior is considered as a categorical variable (user, part-user, non-user), the chi-square test of independence was used for the significance test of the following categorial variables: sex, medical conditions, income, and education level. For the continuous variables, age, body mass index (BMI), baseline activity at B0, and the Shapiro-Wilk test were used to test for normal distribution. The Kruskal-Wallis test was used to test for significance, since the variables did not follow normal distribution. Furthermore, a linear regression was performed for the number of completed courses, reflecting the usage behavior as the continuous variable, while taking into account the aforementioned socio-economic factors.

The Wilcoxon Signed-Rank Test for dependent samples was used to test for significant changes in total activity for both the different app user groups and all participants as a whole. Due to multiple testing, Bonferroni correction was applied, resulting in a significance level of 1.25%. Furthermore, the user feedback regarding the app "Videa bewegt" was analyzed descriptively.

## 3. Results

### 3.1 Patients’ characteristics and app adherence

Of the 264 patients fulfilling the inclusion criteria, 202 (77%) took part in the study. Most of the participants were female (61%) and in the age range of 50–59 (44%). The mean BMI was 30.8 kg/m^2^. 97/202 (48%) of the patients had a BMI >30 kg/m^2^ (obesity class 1), with 49/202 (24%) having a BMI >35 kg/m^2^ (obesity class 2). The leading clinical diagnosis 97/202 (48%) of patients was a psychosomatic disorder (mostly depression), followed by patients with orthopedic diseases (mostly chronic low back pain). 78/202 (39%) received a monthly income between 2,000 and 5,000 €, reflecting a middle class income in Germany [33]. 55/202 (27%) had a high educational level (e.g., university degree or comparable), 90/202 (45%) had a mid-educational level (e.g., high school diploma) and 57/202 (28%) had a low educational level (e.g., lower secondary school graduates). The median total activity at baseline B0 (prior to rehabilitation) accounted to 360 minutes per week, whereas the mean total activity was 513 minutes per week, leading to a right-skewed distribution. 41% of study participants reported a total activity of less than 300 minutes per week, reflecting a mainly sedentary lifestyle.

Of all 202 participants receiving access to the app, 111/202 (55%) did not download it or stopped using the app after the first course; these participants were defined as “non-users”. 33/202 (16%) took part in 2–4 courses and were defined as the “part-user” group. Finally, 58/202 (29%) completed 5 or more courses and belonged to the “user” group. The average number of completed courses over all participants accounted to 3.2 courses. These groups formed the basis for the evaluations regarding socio-economic factors and long-term changes in PAL, which are presented in the following sections.

**Fig 1:**
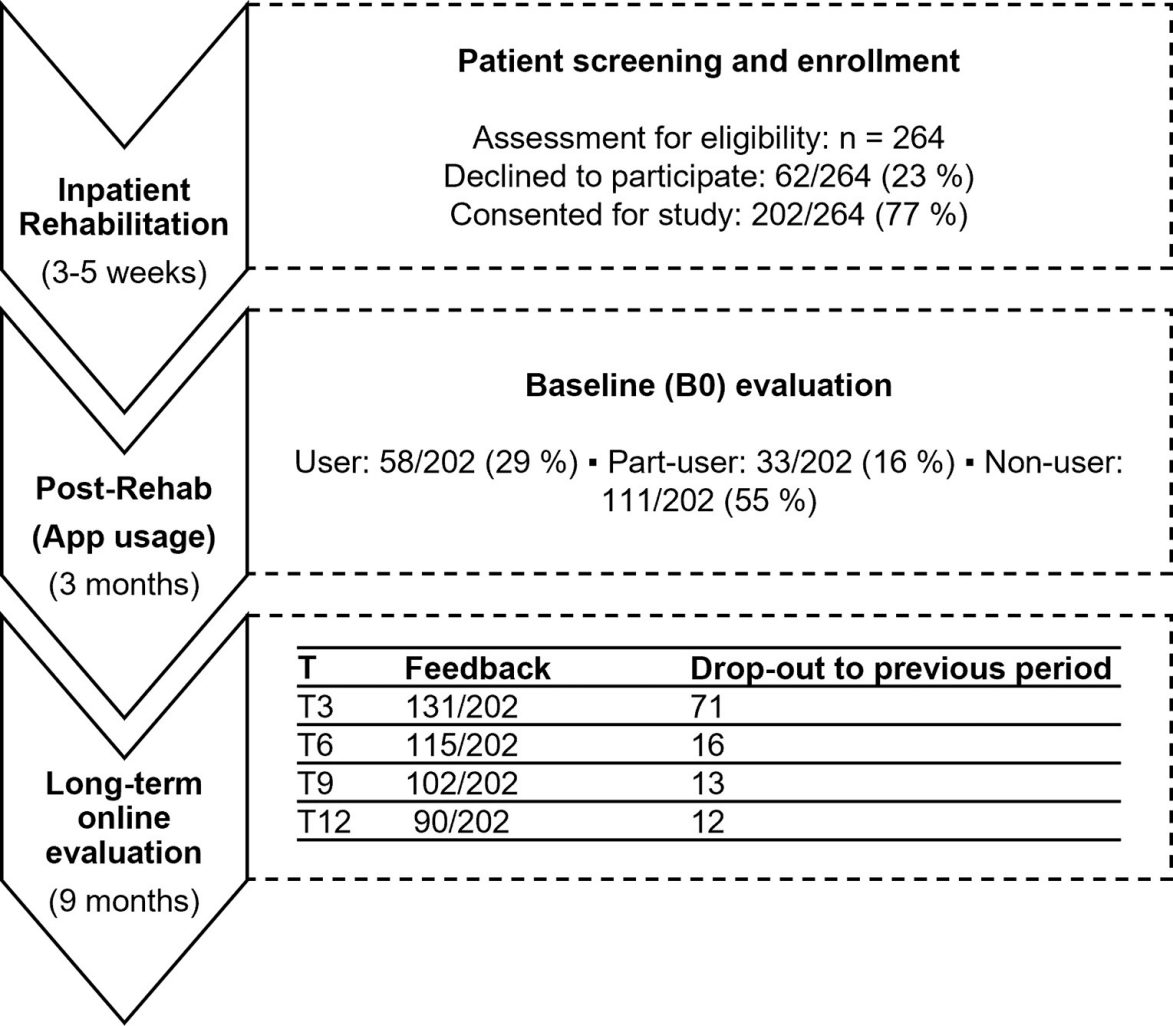
Study enrollment and development of participant number.

Fig 1 visualizes all study phases including the development of participants and drop-outs, whereas Table 1 gives an overview of all assessed socio-economic parameters of the study participants at baseline.

**Table 1:** Characteristics of participants.

Overall [%, (n)]	100	(202)
Sex male [%, (n)]	39	(79)
female [%, (n)]	61	(123)
**Age** <29 [%, (n)] 30–39 [%, (n)] 40–49 [%, (n)] 50–59 [%, (n)] >60 [%, (n)]	1 10 16 44 29	(2) (20) (33) (88) (59)
**BMI** underweight [%, (n)] healthy weight [%, (n)] overweight [%, (n)] obesity class 1 [%, (n)] obesity class 2 [%, (n)] obesity class 3 [%, (n)]	4 20 28 24 11 13	(8) (40) (57) (48) (23) (26)
**Income [€]** low—<1,000 [%, (n)] low/middle—1,000–2,000 [%, (n)] middle—2,000–5,000 [%, (n)] high—5,000–10,000 [%, (n)] very high—>10,000 [%, (n)]	16 35 39 10 0	(32) (70) (78) (21) (1)
**Education level** high level [%, (n)] middle level [%, (n)] low level [%, (n)]	27 45 28	(55) (90) (57)
**FFKa–Total activity [min/week]** < 100 [%, (n)] 100–200 [%, (n)] 200–300 [%, (n)] 300–400 [%, (n)] 400–500 [%, (n)] 500–1000 [%, (n)] >1,000 [%, (n)]	16 14 11 17 8 24 10	(32) (27) (21) (35) (17) (49) (21)
**App user behavior** user [%, (n)] part-user [%, (n)] non-user [%, (n)]	29 16 55	(58) (33) (111)

### 3.2 User feedback

User feedback was collected from 66 participants using a feedback questionnaire on the "Videa bewegt" app after 12 months. 54/66 (83%) stated that they found the app helpful. 48/66 (73%) particularly liked the video-based content, whereas only 23/66 (35%) considered the quiz questions helpful. Regarding the activity tracking, both the tracking of activity minutes with 54/66 (82%) and the number of steps with 48/66 (74%) were rated positively. The community within the app was not considered helpful by 60/66 (91%) of participants. In addition, 22/66 (33%) of the participants had problems using the app. Here, sign-in problems were mentioned particularly frequently. The time required for using the app was rated as reasonable by 41/66 (62%). In addition, 21/66 (32%) stated that they continued using the app after completing the course.

### 3.3 Socio-economic evaluation of app user groups

Table 2 shows the results of the chi-square test to determine significance of the app user behavior depending on the socio-economic factors of sex, medical condition (diagnosis), income, and educational level. The table shows the actual frequency and expected frequency in parenthesis (exp.) as well as the calculated *p*-value. In summary, no significance was observed for any of the investigated factors regarding the app usage behavior.

**Table 2:** Results for user behavior of the application depending on sex, diagnosis, income, and educational level.

	User	Part-user	Non-User
**Sex** Male [act. (exp.)]	15 (16.6)	12 (12.9)	52 (43.4)
Female [act. (exp.)]	43 (35.3)	21 (20.1)	59 (67.6)
***p = 0*,*24***			
**Diagnosis** internal [act. (exp.)] orthopedic [act. (exp.)] psychosomatic [act. (exp.)] ***p = 0*,*24***	5 (9.0) 20 (16.8) 33 (28.1)	7 (5.1) 14 (12.2) 12 (16.0)	19 (17.2) 40 (41.1) 52 (53.8)
	**User**	**Part-user**	**Non-User**
**Income [€]** <1,000 [act. (exp.)] 1,000–2,000 [act. (exp.)] 2,000–5,000 [act. (exp.)] 5,000–10,000 [act. (exp.)] >10,000 [act. (exp.)] ***p = 0*,*75***	16 (11.6) 22 (26.1) 14 (27.6) 6 (7.6) 0 (0.4)	3 (6.6) 13 (14.9) 14 (15.7) 3 (4.3) 0 (0.2)	13 (22.2) 37 (50.0) 48 (52.7) 12 (14.5) 1 (0.7)
**Education level** High level [act. (exp.)] Mid level [act. (exp.)] Low level [act. (exp.)] ***p = 0*,*81***	16 (15.8) 26 (25.8) 16 (16.4)	9 (9.0) 12 (14.7) 12 (9.3)	30 (30.2) 52 (49.4) 29 (31.3)

**Fig 2:**
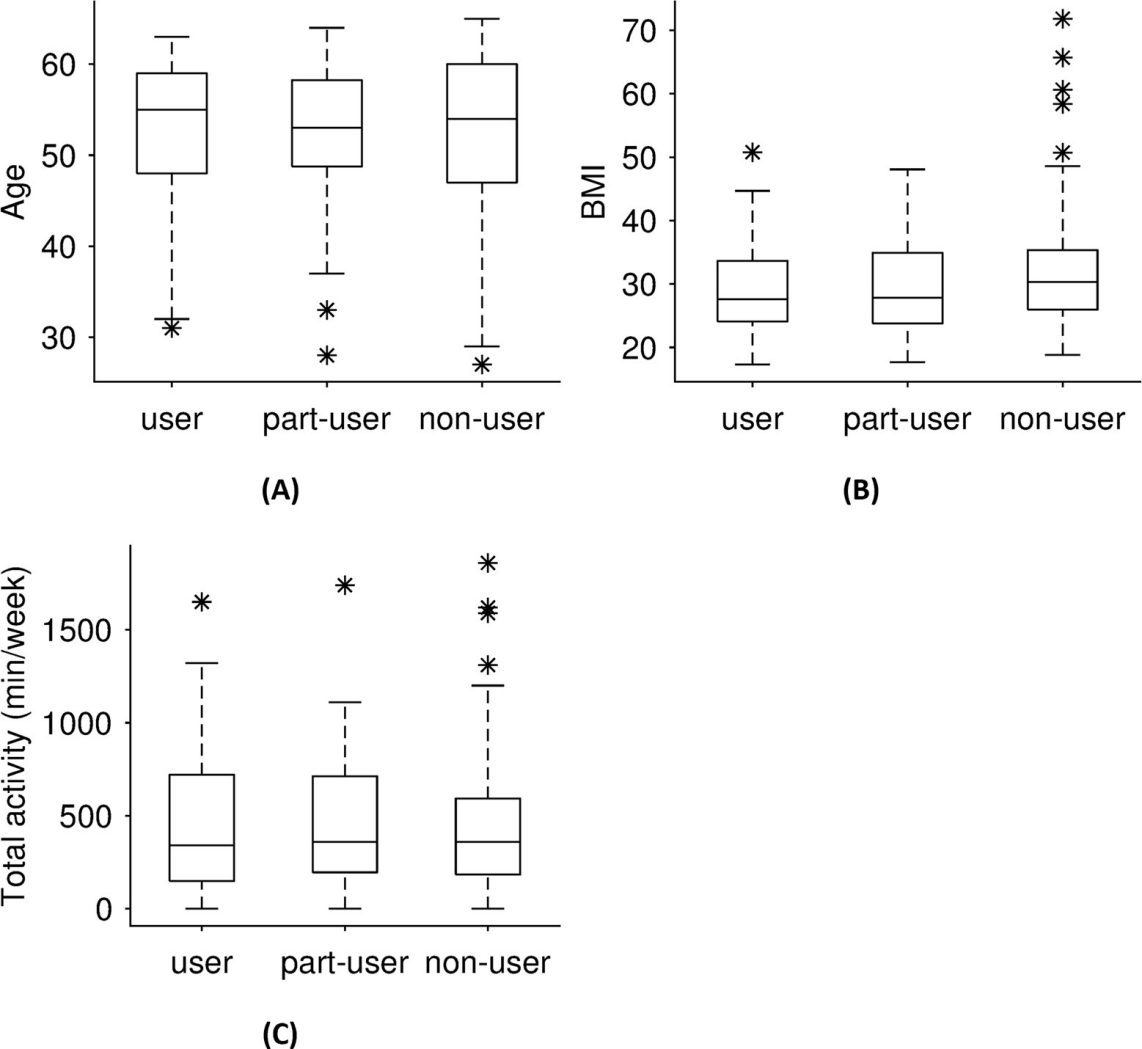
Results for user behavior of the application depending on (A) age, (B) BMI, (C) total activity at baseline B0.

As mentioned in Section 2.5, the significance of the factors age, BMI, and total activity was assessed by performing a Kruskal-Wallis test. Fig 2 shows the median, the 25%/75% quantiles, and the outliers for different user groups for each of the factors examined. No significance was found for any of the parameters examined (p at B0: age = 0.94, BMI = 0.15, total activity = 0.88).

Fig 2A shows no distributional differences regarding the usage behavior and age. Fig 2B indicates that the number of outliers of the body mass index in the non-user group is remarkably higher than for the user and part-user group. This is also reflected in the p-value of the significance test, which was much lower than for the other parameters. Fig 2C suggests a slightly higher median of the total activity at baseline B0 of the user group.

Furthermore, a linear regression was performed on all aforementioned socio-economic factors with continuous (age), categorical (education, income level, medical diagnosis), or binary (sex) variables, as well as the number of completed courses within the app as the outcome variable. Table 3 presents the results for the R^2^- and p-values. Since the model includes an intercept term, it only contains two indicator variables and consequently two p-values, to prevent rank deficiency of the design matrix.

**Table 3:** Results for the linear regression of the completed courses depending on socio-economic factors.

Dependent variable	Independent variable	R^2^	p-value
Number of completed app courses	Sex	0.035	0.02
	Diagnosis	0.013	0.20 | 0.15
	Income	0.100	0.05 | 0.00 | 0.02
	Education	0.001	0.65 | 0.71
	Age	0.001	0.82
	BMI	0.022	0.05
	B0 physical activity level	0.003	0.43

### 3.4 Differences in physical activity level

In the following section, we present the results of the Wilcoxon signed-rank test for the analysis of the total activity change after 3, 6, 9, and 12 months compared to baseline B0 for different app user types as well as the overall group. Therefore, Table 4 summarizes the number of remaining participants *n*, the mean value, the standard deviation SD, the median value, the 25% and 75% quantile of the total activity in minutes per week, as well as the p-value from the Wilcoxon signed-rank test.

While the dropout rate of the total group was 112/202 (55%), dropouts among users and part-users were less frequent at a rate of 33/91 (36%), whereas 79/111 (71%) of non-users were classified as dropouts. While the mean and median values of the total activity of the user group were typically above the corresponding values of the total cohort, the mean and median values of the non-user group were, with exception of T9, always below the total cohort. Regarding the location of the 75% quantile, a slight tendency towards higher values for the user and partial user group could be observed. The analysis showed a significant improvement of the total cohort from B0 to T6. No significance could be demonstrated for the subgroups.

**Table 4:** Results of the Wilcoxon signed-rank test, analyzing the change of the total activity depending on app usage behavior.

		B0	T3	T6	T9	T12
all participants	*n*	202	131	115	102	90
	Mean SD	470 372	527 467	618 509	542 450	357 359
	Median 25% quantile 75% quantile	360 195 640	390 240 660	460 270 790	420 180 780	263 119 461
	**Wilcoxon *p***		**0.1809**	**0.0049**	**0.3088**	**0.0137**
user	*n*	58	56	51	50	42
	Mean SD	496 388	592 564	607 543	456 346	428 458
	Median 25% quantile 75% quantile	392 157 750	397 245 713	445 300 765	409 180 610	273 138 513
	**Wilcoxon *p***		**0.1760**	**0.2202**	**0.5276**	**0.3987**
part-user	*n*	33	24	21	12	16
	Mean SD	462 377	564 483	669 457	716 658	285 226
	Median 25% quantile 75% quantile	360 195 715	435 274 690	450 292 971	580 347 820	227 107 433
	**Wilcoxon *p***		**0.7425**	**0.1865**	**0.1670**	**0.0787**
non-user	*n*	111	51	43	40	32
	Mean SD	459 364	438 311	607 500	598 479	300 236
	Median 25% quantile 75% quantile	360 210 630	330 210 660	570 203 772	435 165 990	249 78 432
	**Wilcoxon *p***		**0.7466**	**0.0232**	**0.1205**	**0.0697**

**Fig 3:**
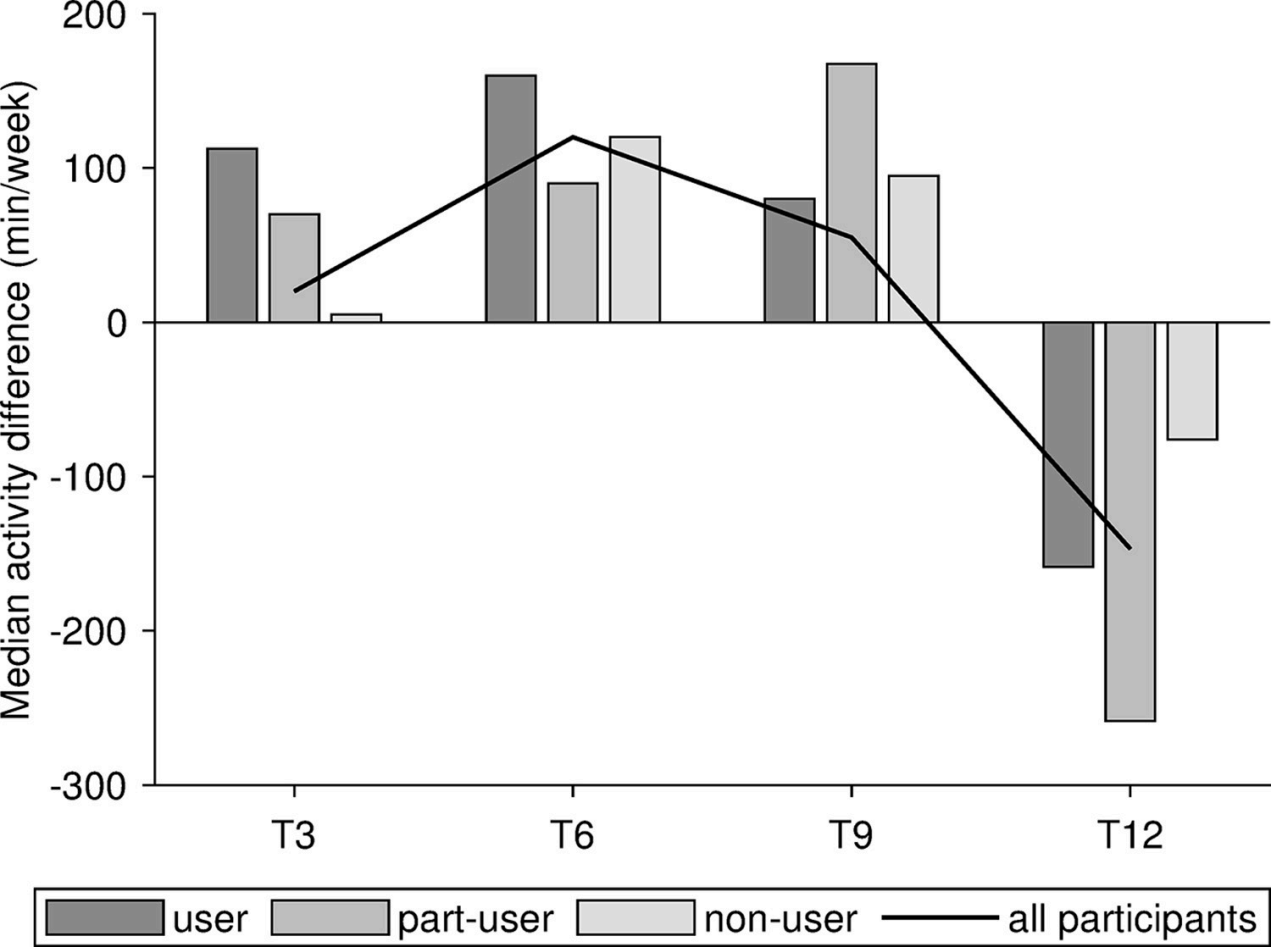
Median activity difference for different months against baseline B0 and median of all participants without considering app user behavior.

To give an overview, Fig 3 displays the median activity difference for different times against the baseline B0. The median activity difference of the different app user types is visualized as a bar chart, where the median activity difference of all participants is plotted as line. Against baseline B0, all groups showed a positive change in their median total activity until T9. The user and part-user groups were more active than the non-user group at T3. After 6 months, the non-user group showed a bigger change in their median activity than the part-user group, though the change was still smaller than in the user group. All groups showed a remaining positive effect of the rehabilitation for 9 months. After 12 months, all groups had a reduction of their median activity compared to baseline B0. The user and part-user groups, who particularly benefitted during the first 6 months, exhibited a large reduction of their total activity.

## 4. Discussion

### 4.1 Study population

Thus far, few studies have addressed the applicability and effectiveness of digital interventions on PAL in a post-rehabilitation setting, since inpatient rehabilitation measures that are provided on a legal basis are available only in selected European countries (Austria, Germany, Switzerland). Our study sample reflects a representative German rehabilitation population characterized by the prevailing (pre-retirement) age group of 50–59 years, with most participants having a non-academic educational level and mostly prevalent chronic health problems associated with an increased risk of early retirement.

In contrast to the general German population, which has an obesity prevalence among adults of 18.1%, 97/202 (48%) of our participants were obese with a BMI >30 kg/m^2^. In a previous study, we found that 84% of said obese rehabilitants were generally interested in digital health technologies, but had little practical experience with freely available mobile health interventions themselves, despite a constantly growing number of available applications [34]. Hence, there still seem to be various barriers to using mobile health applications. In particular, (obese) individuals of lower socio-economic status might be disinclined to using digital interventions because of a lower e-health literacy [25,35]. Previous studies provide contradictory results to this issue. In our study, app usage did not depend on BMI, which underlines that, during inpatient rehabilitation, it was possible to adequately address and motivate obese patients to take part in the study. In addition, BMI and other socio-economic factors had no impact on the primary outcome parameter, the PAL. This finding is in contrast to Western et al., who could not find any evidence in their published meta-analysis about digital interventions in individuals of lower socio-economic levels being effective with regard to PAL [36].

### 4.2 App usage behavior

In contrast to most current publications about studies on the effectiveness of m-health-technologies, our patients underwent an intensive 3-week inpatient rehabilitation program prior to the reported intervention [37–39]. During rehabilitation, they were informed about the importance and need of increasing their PAL according to the WHO guidelines [2]. They were motivated to stay active after rehabilitation by using the app “Videa bewegt” to further support them in their struggle for sustained behavioral change in their daily lives. Not surprisingly, 202/264 (77%) of patients fulfilling the inclusion criteria were interested in the app usage at the end of their rehabilitation and signed the consent form. In contrast to a previous study of Jakob et al. regarding the (documented) app usage behavior, we found no significant difference between the analyzed groups relating to socio-economic factors such as age, medical condition, and education [23]. These findings may be explained by the intense rehabilitation program, which allowed to us to motivate and successfully recruit patients of different socio-economic characteristics. Noteworthily, women were more likely to complete the app program than men. It is known that women both tend to express greater interest in health interventions than men and are easier to convince to test new techniques [30]. In addition, patients with high income tended to be less adherent to the app program. However, this result should be interpreted with caution, as the number of patients in this sample was very small.

There were no significant differences in app adherence behavior regarding baseline PAL and underlying health problems. This also applied for our patients with psychiatric diagnoses and is in contrast to the results of Farvolden et al., who evaluated a 3-month online web program for patients with panic disorders and found that only 1% completed the intervention [40]. Our observations may differ due to the fact that the digital divide has declined during recent years, but may also stem from our special study design [41]. During the preceding intensive inpatient rehabilitation program, it was possible to personally address/motivate the patients and simultaneously introduce them to the app, so that patients could be better persuaded to use it. We suppose that direct communication with patients contributed significantly to the minimization of the digital divide, resulting in us not finding any differences in app usage with regard to the analyzed socio-economic factors. 1/3 patients completed >5/8 courses of the app program, which is a significantly better result compared to the findings previously reported by Meyeritz-Katz et al. [22].

In addition, the percentage of patients who did not complete the program did not differ from previous studies with healthy participants for whom the usage of the App “Videa bewegt” was analyzed, thus emphasizing the usability of the app program not only in healthy, but also in our chronically ill rehabilitants [29,30].

Most non-users reported technical problems and lack of an offline version as reason for non-use (lack of adherence). A more personalized approach in implementing the app could be helpful [42]. Overall, we consider the user and part-user number of 91/202 (45%) quite good for an application-based program. The user feedback (after 12 months) was generally positive, with 54/66 (83%) of feedback-giving participants rating the app "Videa bewegt" as helpful.

### 4.3 Long-term effects on physical activity level

PAL in our total sample at the start of the rehabilitation was 6 h/week and therefore significantly lower than in the study by Frey et al., who found a mean total activity of 9.2 h/week in a systematic sample of the general population of Freiburg, a medium-sized German university town (n = 612), age 20–98 years [32]. There was a strong effect of the inpatient rehabilitation program on the PAL of patients with a significant increase in PAL over all groups (app users and non-users) after 6 months. Median PAL of the total group (total activity minutes/week) was increased by 8.3% after 3 months, 28.0% after 6 months and 17.0% after 9 months in the total study population against baseline. Study participants showed the highest activity level 6 months after rehabilitation, confirming once again that medical rehabilitation is an effective measure for inducing lifestyle changes [43]. Regarding the effectiveness of the “Videa bewegt” app on long-term self-reported PAL, no significant differences between app users and non-users could be observed. The increase in PAL was, however, slightly (but not significantly) more pronounced in app users after 3 and 6 months, which could indicate that the app program may have a positive effect on motivating patients to stay more active in the period of 3–6 months (short-term) after rehabilitation. This observation is supported by several recently published studies, which found small to moderate increases in PA that may be maintained over several months, but which decrease with time [37–39]. In summary, over a period of 9 months post-rehabilitation, all groups were physically more active than at baseline, which is in agreement with our previous study [43]. From this we conclude that the positive effect of the multimodal rehabilitation is the dominant effect on the physical activity level and probably outweighs the influence of the smartphone app. A recently published study with a follow-up time of 6 months showed that using a smartphone-app-maintained PAL following pulmonary rehabilitation in patients with COPD with a significant difference between users and non-users. In contrast to our study, these patients did not increase their activity level on their own after rehabilitation [44]. Their activity remained stable (app intervention) or declined (control group) within 6 months. Other studies investigating the potential effectiveness of m-health technologies on PAL after rehabilitation in other populations are currently under way [42,45].

In contrast to a previous study of ours, there was a decline of PAL below the initial level after 12 months which we interpret as an effect of the Corona-Pandemic. During follow-up time of our study, there was a 7-month lockdown period in Germany during which public life came to a standstill, sports facilities and gyms were closed, and employees were strongly recommended to work from home. An international survey recently confirmed that physically levels substantially decreased in countries affected by COVID-19. Compared to pre-restrictions, overall self-reported PAL declined by 41% (moderate-to vigorous PAL) [46].

Overall, our study supports the results of recently published meta-analyses that m-health technologies can promote an increase of physical activity in chronically ill patients and that the effects of a time-limited intervention may be maintained for several months, albeit with a decrease over time [37–39]. Since current research suggests that the person supervising the patients’ app usage seems to be one of the most important success factors in promoting motivation for and adherence to behavioral change, a combination of mobile health applications and individually tailored programs in a well-defined setting with regular personal feedback might further improve adherence and effectiveness of m-health technologies [42].

### 4.4 Strengths and limitations

One main strength of our study is that we investigated a representative sample of 202 rehabilitants over a period of 12 months under real-life conditions. Another strength is the single-centered study design so that all participants were informed and instructed by the same study personnel. The obtained data on adherence are valid because we had access to the digital database and were thus able to combine socio-economic data with app usage. Nevertheless, most of the collected data (socio-economic factors such as income, PAL) are based on self-assessment, leading to us being unable to rule out patients giving so-called socially desirable answers [47]. One further (unpredictable) weakness is that while the study was planned out before the SARS-CoV-2 pandemic, it was ultimately performed during it, which likely had negative influences on PAL of our study population. In addition, we must consider that other factors, such as psychosocial distress caused by the pandemic etc., could have influenced the behavior of our study population [48,49]. In addition, we cannot rule out seasonal effects when recruiting patients over several months. Not surprisingly, there were more dropouts in the app non-user group. Therefore, we cannot rule out that this group may, in actuality, have performed even worse with regard to PAL.

## 5. Conclusion

To our knowledge, this is the first study that investigated PAL over a period of 12 months in rehabilitation patients using a mobile health application for 3 months post-rehabilitation. Firstly, our study confirms the effectiveness of an inpatient rehabilitation program in this high-burden (mostly obese) patient group. App users as well as non-users successfully increased their PAL after the inpatient rehabilitation. Secondly, almost 50% of our study participants accepted the offer of a digital aftercare program, resulting in them downloading and using the app program; most of them evaluated the app as useful to them, underlining the applicability of the digital after-care program. App usage in this after-care setting did not depend on socio-economic factors, except for the patients’ sex. Thirdly, app users tended to be more active than non-users. In particular, use of the app seemed to contribute to a sustained increase in activity over a period of 9 months. Whether this is a real effect of the app, or the outcome of a higher intrinsic motivation of app users, requires further evaluation in a randomized trial.

## Supporting information

S1 Data The original data is available through the publishing journal. The database holds the socio-economic factors (sex, age, income, education, income, BMI) as well as the overall and sub-categorical physical activity level from the “Freiburg Questionnaire on physical activity level” for all investigated time points. Furthermore, we also provided the number of completed courses from all participants within the database.
(XLSX)
